# The Frequency of Anti-Aquaporin-4 Ig G Antibody in Neuromyelitis Optica and Its Spectrum Disorders at a Single Tertiary Referral Center in Malaysia

**DOI:** 10.1155/2014/568254

**Published:** 2014-11-17

**Authors:** Shanthi Viswanathan, Masita Arip, Norhazlin Mustafa, Jasbir S. Dhaliwal, Norzainie Rose, Sobri Muda, Santhi Datuk Puvanarajah, Mohammad Hanip Rafia, Mark Cheong Wing Loong

**Affiliations:** ^1^Department of Neurology, Kuala Lumpur Hospital, Kuala Lumpur, Malaysia; ^2^Autoimmune Unit, Allergy & Immunology Research Centre, Institute for Medical Research (IMR), Kuala Lumpur, Malaysia; ^3^Department of Radiology, Kuala Lumpur Hospital, Kuala Lumpur, Malaysia; ^4^Department of Radiology, National University of Malaysia, Kuala Lumpur, Malaysia; ^5^Pharmacy Department, Universiti Malaya, Kuala Lumpur, Malaysia

## Abstract

*Background*. In the past the occurrence of neuromyelitis optica in Malaysia was thought to be uncommon and the frequency of anti-aquaporin-4 Ig G antibody was unknown. *Objective*. To evaluate the frequency of anti-aquaporin-4 Ig G antibody (Anti-AQP4 antibody) amongst patients with neuromyelitis optica (NMO) and its spectrum disorders (NMOSD) and the differences between the seropositive and seronegative groups. *Methods*. Retrospectively, 96 patients with NMO/high risk syndromes for NMOSD (HRS-NMOSD) were identified out of 266 patients with idiopathic inflammatory demyelinating disease from a single center hospital based registry. Anti-AQP4 seropositivity was found in 38/48 (79.2%) with NMO, 12/21 (57.1%) with brain involvement at high risk for NMOSD, 12/15 (80%) with transverse myelitis (i.e., 11/15 with relapsing transverse myelitis and one with monophasic transverse myelitis), and 3/7 (42.8%) with relapsing optic neuritis. Sixty-five out of 96 patients, that is, 67.7%, with NMO/HRS for NMOSD were seropositive. Seropositivity was significantly associated with female gender, a higher number of mean relapses, that is, 5.15 ± 4.42 versus 2.10 ± 1.68, longer length of spinal cord lesions, that is, 6.6 ± 4.9 versus 2.9 ± 2.5, vertebral bodies, higher EDSS, 4.5 ± 2.4 versus 2.4 ± 2.6, presence of paroxysmal tonic spasms, and blindness (unilateral/bilateral); *P* < 0.001. Longitudinally extensive cord lesions (contiguous or linear), presence of lesions in the cervical and thoracic regions, and involvement of the central gray matter or holocord regions on axial scans, were also significantly associated with seropositivity; *P* < 0.001. *Conclusion*. NMO and HRS for NMOSD are present in larger numbers than previously thought in Malaysia. More than 2/3rds are seropositive. Seropositive and seronegative NMO/NMOSD have differences that are useful in clinical practice.

## 1. Background and Introduction

Neuromyelitis optica (NMO) is a chronic inflammatory demyelinating disease of putative autoimmune etiology [1]. It mainly affects the optic nerves and spinal cord with the majority of cases having a relapsing disease course [1]. In 2004, a serum autoantibody NMO Ig G, which binds selectively to central nervous system microvessels, pia, subpia, and Virchow-Robin spaces, was identified [1]. The binding sites of this autoantibody were found to co-localize with aquaporin-4 [1]. The anti-aquaporin-4 Ig G antibody (anti-AQP4 Ig G) or NMO Ig G has been found to be highly specific and sensitive for NMO. It has been incorporated into the revised Wingerchuk criteria of 2006 for the diagnosis of NMO [1, 2]. With the aid of this antibody, the spectrum of NMO is now known to be wider than previously recognized [3]. In view of this heterogeneity, a new term, that is, neuromyelitis optica spectrum disorders (NMOSD), was introduced which encompasses patients with recurrent or isolated, longitudinally extensive myelitis or optic neuritis, longitudinally extensive myelitis or optic neuritis associated with systemic autoimmune disease, brain lesions typical for NMO, and some cases of Asian opticospinal multiple sclerosis (OSMS) [2, 3].

Based on previous literature, neuromyelitis optica had been recognized as relatively uncommon in Malaysia. In the past, it was defined as a monophasic disease [4, 5]. Literature on NMO in Malaysia is lacking especially after the revision of the Wingerchuk 2006 criteria [2, 3]. Furthermore, the frequency of the antibody in this group of patients is unknown. Hence, it was decided to pursue the testing of this antibody to find out the frequency of Anti-AQP4 Ig G amongst Malaysian patients presenting with a phenotype suggestive of NMO and other idiopathic inflammatory demyelinating diseases at high risk for neuromyelitis optica spectrum disorder (NMOSD). There have been a number of reports suggesting that treatment response in patients with NMO/NMOSD differs from that used for multiple sclerosis and thus identifying these patients has important therapeutic implications [6–9].

Kuala Lumpur Hospital is a major tertiary referral center with a Neurology service that caters for patients throughout the country. The patient population attending the neurology service is representative of the demographics of the country. To our knowledge this is the first study assessing the frequency of the antibody in patients with IIDDs suggestive of NMO/NMOSD and high risk syndromes for this condition from a single tertiary referral center in Malaysia.

## 2. Objective

The main objective of this study was to evaluate the frequency of anti-AQP4 Ig G in patients with idiopathic inflammatory demyelinating diseases such as NMO and high risk syndromes for neuromyelitis optica spectrum disorder (NMOSD) such as monophasic or multiphasic recurrent transverse myelitis and optic neuritis as well as brain lesions at onset of disease either monophasic/multiphasic, monofocal, or multifocal. A secondary objective was to look at the differences between the seropositive and seronegative groups.

## 3. Methods

This was a hospital based retrospective time series with longitudinal followup looking at consecutive patients who had presented to the Department of Neurology, Kuala Lumpur Hospital, between January 2009 and January 2014 with idiopathic inflammatory demyelinating disease. Data was obtained from case notes retrospectively and clinic follow-ups. This was done by a neurologist (SV). Kuala Lumpur Hospital is a tertiary referral center with a neurology service that caters for patients from all the states within Malaysia.

### 3.1. Inclusion Criteria

Patients were stratified into the following groups barring the presence of anti-AQP4 Ig G antibody (anti-AQP4) testing based on the criteria highlighted below:

1. all patients with idiopathic inflammatory demyelinating disease who fulfilled Wingerchuk criteria of 2006 for NMO excluding anti-AQP4 testing [2],
2. patients at high risk for NMOSD, that is, those with single episode, monophasic or recurrent relapsing optic neuritis (RON/MON) and single (monophasic) episode or recurrent myelitis (MTM/RTM), as outlined by Wingerchuk et al. [2, 3],
3. patients with brain symptoms and signs at onset of disease with magnetic resonance imaging (MRI) of the brain showing demyelinating lesions atypical for multiple sclerosis but typical for NMOSD as described by Pittock et al. [10],
4. patients with monophasic or multiphasic, monofocal or multifocal demyelinating disease involving the brain but did not fulfil the criteria for brain involvement in NMOSD, acute demyelinating encephalomyelitis, or MS.

### 3.2. Exclusion Criteria

1. Those excluded from the study were patients who refused or were unable to consent for inclusion of their data in the study and/or for the purpose of testing for anti-AQP4 antibody. Also excluded were patients with nondemyelinating idiopathic inflammatory diseases involving the brain.
2. Patients diagnosed with symptoms and signs suggestive of multiple sclerosis based on McDonalds's 2005 and 2010 diagnostic criteria [11, 12] were excluded from the study.
3. Patients diagnosed with acute demyelinating encephalomyelitis were also excluded.

### 3.3. Ethical Approval

Institutional Ethics Committee approval was sought from the National Medical Research Register under the auspices of the Ministry of Health of Malaysia and approved for the establishment of a demyelinating diseases registry, for review of available data, and for longitudinal followup. All patients or caregivers gave written informed consent.

Data was collected with regard to demographics, clinical features, neuroimaging, and laboratory parameters. In order to maintain accuracy of interpretation of brain and cord MRI's, neuroimaging at disease onset or the earliest MRI at presentation was used if the former was not available.

Statistical analysis was done with SPSS version 16 looking at absolute medians, means, and descriptive data was expressed as total numbers and percentages. Parametric data was looked at with Student's *t*-test and nonparametric data using the* Chi square* test.

### 3.4. Method of Analysis of Anti-Aquaporin-4 Antibody

The anti-AQP4 test was conducted at the Autoimmune Unit, Allergy & Immunology Research Centre, Institute for Medical Research, Kuala Lumpur. Anti-aquaporin-4 antibodies (AQP4) were determined in the serum of the patients by indirect immunofluorescence (EUROIMMUN IIFT, Germany) on a cell line which had been molecular-biologically modified (AQP4 transfected cells) to produce large quantities of AQP4.

Biochip slides containing AQP4 transfected cells and nontransfected cells (EU-90) were incubated with diluted patient samples. In the case of positive reactions, specific antibodies of the classes IgA, Ig G, and IgM would bind to the antigens. In a second step, the attached antibodies are stained with fluorescein-labelled anti-human antibodies and made visible with the fluorescence microscope.

### 3.5. Clinical Material

A total of 266 patients were identified from the database. These patients had visited the Neurology Department at Kuala Lumpur Hospital between January 2009 and January 2014. Median duration of followup was 3 years. Out of the total number of patients in the database, there were 58 patients with neuromyelitis optica. In addition, there were 22 subjects with brain involvement at onset of disease suggestive of NMOSD. Twenty of whom went on to develop optic neuritis or transverse myelitis or both on longitudinal followup. Nineteen patients had transverse myelitis alone of which 14 had relapsing transverse myelitis (RTM) and 5 had monophasic transverse myelitis (MTM). Four of these patients with MTM were excluded due to incomplete data. Seven patients had relapsing optic neuritis and three had monophasic optic neuritis. One hundred and thirty-two patients had multiple sclerosis and twenty were diagnosed to have acute demyelinating encephalomyelitis and both groups were excluded from the study. Five patients out of the 266 patients in the database had monofocal monophasic disease affecting the brain stem not typical of multiple sclerosis or NMO of which 3 patients had incomplete data and were excluded. These patients were still considered at high risk for NMOSD as all other causes had been excluded. Testing for anti-aquaporin-4 antibody was done on 48 patients with NMO, 21 with NMOSD (brain involvement at onset), 15 with transverse myelitis, 7 with relapsing optic neuritis, 3 with monophasic optic neuritis, and 2 in patients with monophasic, monofocal brain disease at onset (whose brain MRI lesions did not look like those described by Pittock et al. but still were possibly at high risk for NMOSD). 10 patients with NMO and one with brain NMOSD did not have anti-aquaporin-4 testing done as they were lost to followup or had incomplete data.

## 4. Results

Patient demographics are shown in (Table 1). Overall, the majority of patients were females (88.5% versus 11.5%). The Malays were the predominant racial group affected, 47/96 (49.0%), followed by the Chinese, 41/96 (42.7%), Indians, 6/96 (6.2%), and other indigenous groups such as Ibans (1.1%) and Bajaus (1.1%). Relapsing remitting disease (90/96, 93.8%) was the commonest disease course with progressive course (1%) and monophasic disease (5.2%) being rare. Median age at onset was 30 ± 11.5 years, median duration of disease was 2.0 ± 0.99 years (range 1 to 6 years), and median EDSS on the last followup was 3.0 ± 2.48, (range 0 to 9). Median duration between first and second attack was 0.5 ± 1.1 years (range 1–7 years). Median annualized relapse rate was 1.0 ± 0.73 (0 to 3.4). Clinically, transverse myelitis was the commonest initial presentation followed by optic neuritis in this mixed group (Table 1).

Overall, 65 subjects or 67.7% tested positive out of 96 patients with idiopathic inflammatory demyelinating disease at high risk for NMO/NMOSD. In the NMO group, 38/48 (79.1%) subjects tested positive for anti-AQP4 antibody. Out of the 21 subjects with brain involvement at onset suggestive of NMOSD, 12 (57.1%) were positive for the antibody (Table 2).

**Figure 1:**
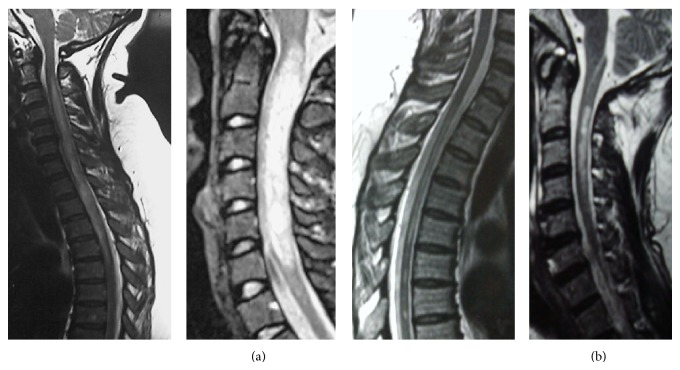
T2WI (sagittal view) longitudinally extensive contiguos cord lesion involving the cervicothoracic cord ((a) extreme right and center) and longitudinally extensive linear cord lesion (a) extreme left. (b) Longitudinally linear and contigous cord lesion.

In the transverse myelitis group, 12 (80%) patients were antibody positive of which 11 patients had relapsing myelitis (Table 2). Out of the 11 with relapsing myelitis who were positive, the majority had either longitudinally contiguous lesions (6/11), linear lesions (3/11), and finally a combination of longitudinally linear and contiguous lesions (1/12) (Figure 1). One patient with short segment relapsing myelitis was also positive for anti-AQP4 antibody. She initially presented with paroxysmal tonic spasms, weakness of left upper limb, and a short cord cervical lesion of 2 vertebral segments (cervical 1 to 2) in length and was seronegative. At first she was started on beta-interferon I-b but after one and a half years she experienced another cord relapse with a longitudinally extensive cervical cord lesion from cervical vertebrae 2 to 5 and subsequently after the second relapse a thoracic vertebrae 6 (T6) to 10 (T10) lesion and became anti-AQP4 antibody positive. Interferons were substituted with immunosuppressants. Brain MRI repeatedly showed nonspecific white matter lesions. Another patient with monophasic longitudinally extensive contiguous cord lesion involving the entire spinal cord was also seropositive; she was started on immunosuppressants and maintenance steroids and remained relapse free till today. However, at year 5 on immunosuppressants she developed neutropenia necessitating an interruption in azathioprine.

Only three patients (42.8%) with relapsing optic neuritis were positive for anti-AQP4 antibody out of 7 patients. Three patients with monophasic optic neuritis were seronegative. Out of the three seropositive patients, one went on to develop an asymptomatic short cord lesion on surveillance magnetic resonance imaging (MRI) after 2 years. None of the patients with monophasic or monofocal demyelinating disease of the brain were anti-AQP4 antibody positive. We are still following up these patients to see their outcome.

We also looked at the association between the presence of the anti-AQP4 antibody and certain clinical and neuroimaging parameters. Seropositivity was significantly associated with a higher current mean EDSS (4.5 ± 2.4 versus 2.4 ± 2.6, *P* < 0.001) compared to seronegatives. It was also significantly associated with an increased mean number of relapses, that is, 5.15 (±4.42) versus 2.10 (±1.68), in the seronegative group, *P* = 0.001. Furthermore, seropositive NMO/NMOSD was significantly associated with paroxysmal tonic spasms, *P* = 0.004, and longer length in spinal cord lesions, that is, 6.66 (±4.9) versus 2.90 (±2.5) vertebral segments, *P* < 0.001. Blindness in one or both eyes and poor visual acuity were significantly seen in seropositive patients rather than seronegative patients (Tables 3 and 4). NMO/NMOSD patients with longitudinally extensive cord lesions of contiguous or linear nature with or without fragmentation/interrupted lesions were significantly associated with being anti-AQP4 antibody positive rather than being negative, *P* < 0.001. Seropositive patients had significantly more lesions in the cervical, thoracic, and cervicothoracic cord regions, *P* < 0.001. More seropositive patients had cord atrophy; however, this did not achieve statistical significance, *P* = 0.056. Holocord or central gray matter lesions were significantly associated with seropositivity (*P* < 0.001). Seronegative patients had significantly lesser number of cord lesions as compared to seropositive patients (0.96 versus 1.17), *P* = 0.025 (Table 3). On the other hand, seropositivity and seronegativity did not discriminate between the different ethnic races or the type of clinical presentation at onset probably due to the small sample sizes. The analysis also showed seropositivity was not significantly associated with time to conversion to NMO/NMOSD, age at onset and duration of disease (Table 4). For those patients who were seronegative in our study we plan to closely follow their clinical phenotype and repeat their antibody testing for anti-AQP4 in the future.

## 5. Discussion

This study attempted to determine the frequency of anti-AQP4 Ig G amongst Malaysian patients presenting with idiopathic inflammatory demyelinating disease (IIDDs) at high risk for NMO/NMOSD. In our study, 65 out of 96 patients, that is, 67.7%, were positive. Historically, studies conducted in Malaysia, from the 1980s, found very few patients presenting with neuromyelitis optica [4, 5]. This could be due to the fact that NMO, according to the original definition used by Devic, was considered to be a monophasic disease [13]. At that time, relapsing forms of severe optic nerve and cord disease were characterized as opticospinal multiple sclerosis regardless of cord length and most were of Chinese origin [4, 5].

However with the revised Wingerchuk criteria of 2006, relapsing forms have been recognised and the spectrum of NMO has been found to be wider with the discovery of the anti-AQP4 antibody for NMO [1–3]. In our study, the majority of patients with an opticospinal presentation had neuromyelitis optica with a longitudinally extensive cord lesion and were seropositive. The diagnosis of opticospinal multiple sclerosis had been made initially in some cases but was revised with the recognition of the significance of the longitudinally extensive cord lesions. In some cases the diagnosis had to be reviewed after obtaining the initial MRI spine at presentation showing such a lesion. Furthermore in other patients, the persistently normal looking brain MRI in the absence of typical lesions for multiple sclerosis on longitudinal followup and development in some cases of typical lesions for NMO/NMOSD aided by seropositivity led to better characterization and diagnosis. We also noted that cord lesions tend to change with time, disease activity, and treatment. So if patients with opticospinal presentation were reviewed without the initial MRI of the spine at onset, there was a possibility of classifying them as multiple sclerosis rather than neuromyelitis optica. In a study from Japan, out of 19 Japanese patients with opticospinal multiple sclerosis (OSMS) and 13 with classical multiple sclerosis tested for NMO-Ig G, 14 patients with OSMS and two patients with classical MS were positive [14]. The two MS patients who were positive had features suggestive of NMO/NMOSD with longitudinally extensive cord lesions and brain involvement atypical for MS, respectively. In their study, NMO seropositive patients frequently had longitudinally extensive transverse myelitis (93% versus 57%) and more severe visual deficits in at least one eye (50% versus 0%) [14]. Furthermore, this similarity can be seen in our own analysis. In our study despite the ethnic differences, seropositivity was associated with poorer visual outcome and longer length of cord lesions. This disease in our country is not so dissimilar to NMO/NMOSD in other Asian /African ethnic cohorts.

We noted that, among the patients who fulfilled the Wingerchuk 2006 criteria for NMO, 79.2% were positive. In the other IIDD groups at high risk for NMO/NMOSD, 11/15 (73.3%) patients with relapsing transverse myelitis and 1/15 with monophasic disease were positive as were 3/7 (42.8%) with relapsing optic neuritis. None of the patients with poorly characterized idiopathic isolated monofocal monophasic demyelinating disease of the brain (brainstem) were positive. The majority of the patients in our study, 69/94, had longitudinally extensive cord lesions at onset of disease either longitudinally contiguous (44), linear (11), both (5), or longitudinally linear and contiguous with patchy interrupted and fragmented lesions (9) (Table 1). Seropositive NMO/NMOSDs in our study were found to be significantly associated with a mean length of the spinal cord lesions of more than 6 vertebral segments (6.6 versus 2.9, *P* < 0.001), holocord and central gray matter involvement, and a tendency for the cervical, thoracic, and cervicothoracic cord to be involved as compared to seronegative patients. We found seropositivity to be significantly associated with the presence of a longitudinally extensive cord lesion (LESCL) whether linear or contiguous in nature. Occasionally, this LESCL extended to the medulla. Seronegative patients had significantly lesser number of cord lesions compared to seropositive patients, that is, 0.96 versus 1.16, *P* = 0.025.

In the seronegative group, a number of patients had short cord segments of between 2 to 3 vertebral segments and hence the mean cord length in the seronegative group was borderline at 2.9 vertebral segments. A nearly equal number of patients with opticospinal or brain involvement at onset of disease who were seropositive and seronegative were found in our study. In the past, some of these patients with opticospinal disease might have been characterized as opticospinal multiple sclerosis.

Regionally, a study by Lu et al. compared 29 patients with NMO to 22 patients with MS and found that NMO patients have significantly more linear lesions than MS patients, that is, 48.3% versus 0%, *P* < 0.001 with these lesions distributed in the spinal cord, medullospinal region, and medulla [15]. In their study, longitudinally extensive cord lesions were also seen more frequently in NMO than MS, that is, 72.4% versus 22.7%, *P* < 0.001 [15]. Studies from Thailand and Taiwan have demonstrated similar observations in their cohorts with LESCLs and seropositivity [16, 17]. In fact, Wingerchuk et al. found the presence of a longitudinally extensive cord lesion extending more than 3 vertebral segments to be the most reliable feature in the diagnosis of NMO [1, 2].

In a review of literature by Jarius and Wildemann, the frequency of anti-AQP4 antibody in patients with longitudinally extensive transverse myelitis (LETM) ranged between 0% and 100% with a median of 53.3% [18]. Though not all of the studies reviewed differentiated between monophasic and recurrent LETM, a higher frequency of antibody positivity was seen in patients with LETM. These results are very comparable to the rates that we got in our relapsing transverse myelitis cohort, that is, 80%. One patient with monophasic transverse myelitis was also seropositive and had a longitudinally extensive contiguous cord lesion affecting the entire spinal cord.

We found an equal number of patients with NMO/NMSOD with short segment myelitis who were seropositive and seronegative. This occurred usually at the onset of the disease. Therefore, in patients with opticospinal or short segment spinal lesions we still feel it may be worthwhile to do the anti-AQP4 antibody test. This is especially so if the brain MRI is not in keeping with MS and is persistently so even when repeated and has some atypicality resembling NMO/NMOSD such as the presence of paroxysmal tonic spasms. Later on, their lesions lengthened to become longitudinally extensive, an observation of ours not reported in the outcome. In the overall cohort, 12 patients had short segment myelitis, less than 3 vertebral segments.

Similarly, if we compare our results in patients with RON to a study from Jarius and Wildemann, in a large cohort, the antibody was found in three out of 89 patients with a single attack of optic neuritis and in five out of 50 with relapsing ON (10%) using cell based assays [18]. Our rates may be higher due to the smaller sample size.

NMO antibody seropositivity has been tested within a diverse population in the West and in Asia. Regionally, a study from Thailand found seropositivity in 18 out of 23 patients (78%) who fulfilled Wingerchuk's criteria for NMO which is very similar to the rate in our study and all the more interesting since Thailand is geographically to the north of Malaysia [1, 2, 16]. Unni N et al. from India reported 80.9% of NMO patients and 54.5% of patients with longitudinally extensive cord lesions to be seropositive [19]. Y. Long et al. from China reported 88.6% of NMO patients, 4.3% with MS, 30.8% with optic neuritis, and 51.7% with longitudinally extensive transverse myelitis to be seropositive [20]. In western literature, though MS is far more common than NMO/NMOSD, Jarius et al. found a seropositivity of 61.1% (22 of 26) among NMO patients as defined by the 1999 Wingerchuk criteria, with 35 of the 36 having long cord lesions [18].

More recently, the same review by Jarius and Wildemann highlighted the heterogeneity in sensitivities for anti-AQP4 antibody existing amongst different studies when different assays were used [18]. This occurred not only due to technical difficulties with the assays but also due to the smaller sample sizes. In their review, when adequate sample sized studies were included, that is, >40 NMO patients, the sensitivities were in the realm of 73.58%, similar to what we see in our study for patients with NMO. This brings home the fact that rates seem to be fairly comparable amongst different ethnic groups in different regions provided there is heightened awareness, the diagnosis is made carefully (i.e., to exclude patients with multiple sclerosis), samples have good effect sizes, and more importantly if a sensitive assay is used taking into account the treatment effect and assessment coinciding with disease remission.

Other salient differences were that seropositive patients were more likely to go blind, experience tonic spasms, have more relapses, and have a higher EDSS. Therefore, when faced with the seropositive NMO/NMOSD patient, therapy needs to be more aggressive as patients have a higher chance of disease worsening due to tendency to relapse more (Table 4). A large multicentre study from Germany [21] and a single center study from Thailand [22] described similar findings. In their cohort of patients, those who were seropositive were more likely to have poorer visual acuity, longer cord lesions, and in the former study to relapse more. In these two studies, females were more affected. In our cohort of patients we also noted the significantly higher female preponderance. Our ratio for female preponderance in the seropositive group was 12 : 1. A similar observation was also noted by Lin et al. in their sample of 57 Chinese patients where the ratio was noted to be 8.5 to 1 [23]. This high female predisposition has already been well described in Caucasian patients where NMO/NMOSD is nine times more prevalent in women than in men [3].

## 6. Conclusion

This study showed that patients with the NMO/NMOSD phenotype do exist in our country in larger numbers than was previously estimated and are not dissimilar from descriptions elsewhere. This is the first study to characterize the frequency of this group of patients in multiethnic Malaysia. When diagnostic criteria is applied appropriately with sensitive and specific assays for anti-aquaporin 4 antibody, it is evident across different ethnic groups around the world that similarities in clinical features, presentation, and neuroimaging exist. The majority of the patients with NMO/NMOSD are anti-AQP4 antibody positive with rates comparable to those around the region. However, we acknowledge the limitations of the study, that is, retrospective nature, descriptive study with small sample size especially within the optic neuritis, transverse myelitis, and demyelinating brain disease at onset groups, referral bias being a tertiary referral centre, and lack of comparison between the NMO/NMOSD and MS groups, as well as the need to use more sensitive and specific assays. We are hoping to address these issues in future studies. With better diagnostic ability to characterize these patients, it will have important therapeutic and economic implications.

## Figures and Tables

**Table 1:** Demographic and clinical characteristics of patients with neuromyelitis optica.

Parameter	Number	Percentage/%
Age at onset (years)	30 (12–52)	
Median (range)		
Gender		
Female	85	88.5
Male	11	11.5
Ethnic origin		
Malays	47	49.0
Chinese	41	42.7
Indians	6	6.2
Ibans	1	1.1
Bajaus	1	1.1
Duration of followup	3.5 (1–12)	—
Median (range)		
EDSS on the last clinic visit	3.0 (0–9)	—
Median (range)		
Commonest clinical features at onset of disease		
ON	35	36.5
Spinal cord	40	41.7
Brainstem/±thalamic &/hypothalamic involvement	15	15.6
Subcortical white matter	4	4.2
Type of cord lesion for all		
Longitudinal contiguous	44	45.8
Longitudinal contiguous & interrupted	9	9.4
Longitudinal Linear	12	12.5
Longitudinal Linear & interrupted	2	2.1
Longitudinal Linear & contiguous	3	3.1
Short segment	12	12.5
No Lesion	13	13.5
Multiple short segments	1	1
Length of Vertebral segments		
1–<3	14	14.6
3.5 to 5	27	28.1
5.5 to 7	23	24.0
7.5 to 10	10	10.4
>10	9	9.3
Nil	13	13.5
Disease course		
Relapsing remitting	90	93.7
Monophasic	5	5.2
Progressive	1	1.0
Diagnosis		
NMO	48	50
TM (HRS for NMOSD)	15	15.6
RTM	14	14.5
MTM	1	1.1
ON (HRS for NMOSD)	10	10.4
RON	7	7.3
MON	3	3.1
Brain involvement at onset	21	21.8
(fulfilling descriptions by Pittock et al. [10]) at high risk for NMOSD		
Other monophasic, monofocal demyelinating disease affecting the brain	2	2.1
Oligoclonal bands		
Positive	6	8.6%
Negative	64	91.4%

Abbreviations: NMO: neuromyelitis optica; TM: transverse myelitis; HRS: High risk syndrome; NMOSD: neuromyelitis optica spectrum disorder; RTM: relapsing transverse myelitis; MTM: monophasic transverse myelitis; RON: relapsing optic neuritis; MTM: monophasic transverse myelitis; EDSS: expanded disability status scale; ON: optic neuritis.

**Table 2:** Frequency of anti-AQP4 antibody in patients with neuromyelitis optica and high risk syndromes for neuromyelitis optica.

Parameter	Anti-Aquaporin 4 antibody positive/%	Anti-aquaporin 4 antibody negative/%	Total
Neuromyelitis optica	38 (79.2%)	10 (20.8%)	48
Myelitis			
Relapsing	11 (73.3%)	3 (20%)	15
Monophasic	1 (6.7%)	0	
High risk syndrome for brain involvement at onset of disease suggestive of neuromyelitis optica spectrum disorder	12 (57.1%)	9 (42.8%)	21
Optic neuritis			
Relapsing	3 (42.8%)	4 (57.1%)	7
Monophasic	0	3 (100%)	3
Monofocal, monophasic demyelinating disease involving the brain (mainly brainstem) not typical of MS or NMO	0	2 (100%)	2

Total	65 (67.7%)	31 (32.3%)	96

Abbreviations: Anti-AQP4 antibody: anti-aquaporin 4 antibody; NMO: neuromyelitis optica; MS: Multiple sclerosis.

**Table 3:** Showing relationship between seropositivity in NMO/NMOSD/High risk syndromes for NMOSD and clinical parameters.

Parameter (Mean)	Seropositive	Seronegative	*P* value, <0.05 is significant
Age at onset	32.9 ± 11.8 years	30.3 ± 10.8 years	0.25
Duration of disease	2.6 ± 1.0 years	2.3 ± 0.8 years	0.23
Number of relapses	5.15 ± 4.4	2.10 ± 1.7	0.001
Annual relapse rates	1.17 ± 0.7	0.91 ± 0.8	0.015
EDSS	4.5 ± 2.4	2.4 ± 2.6	<0.001
Length of spinal cord lesion	6.66 ± 4.9	2.90 ± 2.5	<0.001
Number of cord lesions	1.17 ± 0.96	0.96 ± 0.93	0.025

Abbreviations: EDSS: Expanded disability status scale.

**Table 4:** Showing the differences between Anti-AQP4 seropositive and seronegative cohorts.

	Anti-AQP4 positive					Anti-AQP4 negative					*P* value, <0.05 is significant	
Diagnosis	NMO	RTM	RON/MON	MTM	NMOSD—Brain	NMO	RTM	RON/MON	MTM	NMOSD—Brain	AQP4 +ve/*P* value	AQP4 −ve/*P* value
					inv at onset					inv at onset.		
Gender												
Female	37	10	3/0	1	9	7	3	3/2	0	9	0.02	0.384
Male	1	1	0/0	0	3	3	0	1/1	0	0		
Race												
Malays	19	1	2/0	0	5	7	1	3/2	0	5	0.121	0.935
Chinese	17	7	1/0	1	7	1	2	1/1	0	3		
Indians	2	3	0/0	0	0	1	0	0/0	0	0		
Bajaus	0	0	0/0	0	0	0	0	0/0	0	1		
Ibans	0	0	0/0	0	0	1	0	0/0	0	0		
Blindness												
Yes	26	0	1/0	0	2	5	0	0/2	0	1	0.003	0.044
No (one/both eyes)	12	11	2/0	1	10	5	3	2/0	0	8		
PTS												
Yes	27	10	0/0	1	5	5	2	0/0	0	2	0.004	0.387
No	8	0	3/0	0	5	3	1	2/0	0	3		
Cord atrophy												
Yes	21	9	0/0	1	4	3	1	0/0	0	2	0.056	0.299
No^§^	17	2	3/0	0	7	4	2	2/0	0	6		
^§^Type of cord lesion												
Long Cont	21	6	0	1	9	3	2	0/0	0	2	<0.001	0.220
Long Cont & Int	4	0	0	0	1	3	0	0/0	0	0		
Long Linear	9	3	0	0	0	0	0	0/0	0	0		
Long Linear & Int	1	0	0	0	0	1	0	0/0	0	0		
Long Conti & Linear	1	1	0	0	0	1	0	0/0	0	1		
SS	2	1	0	0	2	2	1	0/0	0	2		
^§^Site of axial cord lesion												
Holocord	19	4	0	1	6	4	2	0/0	0	4	<0.0001	0.059
Central Gray matter	19	7	0	0	5	5	2	0/0	2	4		
Periphery of cord	0	0	0	0	0	0	0	0/0	0	0		
Partial diameter of cord	0	0	0	0	1	0	0	0/0	1	1		
^§^Site of cord lesion												
Cervical	11	3	1/0	0	7	4	1	0/0	0	3	<0.0001	0.116
Thoracic	5	2	0/0	0	1	1	1	0/0	0	0		
Cervico-thoracic	22	6	0/0	0	3	4	1	0/0	0	2		
Thoracolumbar	0	0	0/0	0	1	0	0	0/0	0	0		
Whole spine	0	0	0/0	1	0	0	0	0/0	0	0		

Abbreviations: NMO: Neuromyelitis optica; NMOSD: neuromyelitis optica spectrum disorder; RON: Relapsing optic neuritis; RTM: Relapsing transverse myelitis, MON: Monophasic optic neuritis; MTM: Monophasic transverse myelitis; Anti-AQP4 antibody: Anti Aquaporin 4 antibody, PTS: paroxysmal tonic spasms; Long Cont: Longitudinally contiguous; Long Linear: Longitudinally Linear; Long Cont & Int: Longitudinally contiguous and interrupted; Long Linear & Int: Longitudinally linear and interrupted; Long Cont & Linear: Longitudinally contiguos and linear, SS: short segment.

^§^Patients with no lesions were not included in the table.

^*^Multiple short segment lesions were left out of the table—only one patient in the seropositive group.

For parameters PTS, cord atrophy, type of cord lesion, site of axial cord lesion and site of cord lesion, only available data was included. Patients with no cord lesions and those with monofocal, monophasic demyelinating diseases with onset in the brain but atypical for MS or NMO and were seronegative were excluded and thus the total number of patients will not be 96.

## Abbreviations

NMO: Neuromyelitis optica
NMOSD: Neuromyelitis optica spectrum disorder
RTM: Relapsing transverse myelitis
MTM: Monophasic transverse myelitis
RON: Relapsing optic neuritis
Anti-AQP4: Anti-aquaporin-4 antibody
VS: Vertebral segments
LESCL: Longitudinally extensive spinal cord lesions
SV: Refers to a neurologist, Shanthi Viswanathan.
